# Where there is a will, there’s a way: Job search clarity, reemployment crafting and reemployment quality

**DOI:** 10.3389/fpsyg.2022.1061847

**Published:** 2022-12-05

**Authors:** Xuan Liu, Zhenhao Wu, Kailun Zeng

**Affiliations:** School of Economics and Management, Nanjing Tech University, Nanjing, China

**Keywords:** job search clarity, reemployment crafting, reemployment quality, reemployment context, rural migrant worker

## Abstract

Job hunting is regarded as a self-regulatory process. However, few studies have examined the mechanism underlying the job search goal-performance relationship from the perspective of the self-regulatory behavior of reemployment crafting (RC). Therefore, the purpose of this study was to examine the mediating role of RC in the relationship between job search clarity (JSC) and reemployment quality (RQ) and the moderating role of the reemployment context. A three-wave study was conducted among 295 rural migrant workers who had experienced unemployment to successful reemployment in China. Model 4 and Model 9 from SPSS macro PROCESS were used to test the moderated mediation model. The findings indicated that (1) JSC was positively correlated with RQ; (2) seeking resources (SR) and seeking challenging demands (SCD) fully mediated the relationship between JSC and RQ; (3) supportive environment (SEn) and challenging environment (CEn), independently, have moderating effect on the relationship between JSC and SR, as well as the relationship between JSC and SCD; and (4) the mediating effect of SR as well as SCD was significant and greater when SEn and CEn were both at high levels. This study contributes to goal-setting theory and highlights the important roles of RC and the reemployment context.

## Introduction

Currently, the new mutant strain of COVID-19 is spreading faster and in a more insidious manner (Gao et al., 2022), which makes pandemics more difficult to prevent and control, and although the “dynamic zero COVID-19” policy has been adhered to in China, domestic outbreaks have occurred occasionally. The downwards pressure on the Chinese economy has further intensified due to a combination of recurring pandemics, market downturns, supply chain tensions, and rising raw material prices (Liu et al., 2022), and some small and medium-sized enterprises have inevitably been hit. According to the Chinese State Administration of Market Regulation, in 2021, a total of 13.238 million market entities of all types were written off nationwide, including 3.491 million enterprises and 9.619 million individual entrepreneurs (Sun, 2022). As a result, many enterprises had to reduce their business scale and lay off employees to maintain basic organizational operations, ultimately having a tremendous impact on urban employment. Rural migrant workers (migrant workers) are a group of people who have emerged in the process of China’s new urbanization. They move from rural to urban areas to work mainly at ground-level positions in manufacturing and service industries and have become an indispensable force in the modernization and development of China’s cities (Ming and Wang, 2015). According to the National Bureau of Statistics of China, the number of migrant workers had exceeded 290 million by the end of 2021 (National Bureau of Statistics, 2022). They are a key link in the urban labor supply chain, and the economic and social functioning of cities would be paralyzed without them. Moreover, the Chinese government has been emphasizing the need to expand the size of the middle-income group, while migrant workers, with lower incomes and a lack of social security, are in a relatively disadvantaged position among the urban population and are more vulnerable to the impacts of the pandemic; many have lost their jobs as a result. Therefore, how to find reemployment, especially a satisfying and well-matched job in the post-COVID-19 era, has become a common concern not only for employment management agencies but also for migrant workers.

An effective job search can increase the probability and speed of (re)employment is one of the most consistent findings in the job search literature (Kanfer et al., 2001), and job search normally begins with the identification of a goal, which in turn shapes job search behaviors, and ultimately ends with goal achievement. Job search clarity (JSC), which was first proposed by Wanberg et al. (2002) based on goal-setting theory in their study of the unemployed, is “the extent to which job seekers have clear job search goals and have a clear idea of the type of career, work, or job desired.” Côté et al. (2006) argued that JSC is not only job seekers’ clarity of their job search goals and orientations but also their clarity on how to find a job and how long it takes to find a job. JSC has an important influence on job search behavior and outcomes. Existing studies have demonstrated that JSC can promote job search intensity and job search behaviors (Zikic and Saks, 2009; Bao and Luo, 2015; Liu and Zhang, 2017) and help individuals select appropriate job search strategies (Kuron, 2020), consequently contributing to job search success, but there is a lack of empirical evidence on whether JSC helps unemployed migrant workers find jobs that they feel matched and satisfied with.

Goal-setting theory posits that the process from goal setting to goal achievement is self-regulatory, and factors such as goal orientation, effort, persistence, and task strategy play the mediating roles in the path from goal to performance (Latham and Locke, 1991). Kanfer et al. (2001) maintained that job search is a self-regulated, dynamic goal-directed process in which job search goals are achieved by setting goals and undergoing continuous self-regulation. Therefore, effective self-regulation is essential for individuals finding reemployment. RC, theoretically grounded on the job demands-resources model and job crafting literature, is referred to as the changes the unemployed make in their job search resources and job search demands when trying to find reemployment (Hulshof et al., 2020a). Job crafting is a self-regulatory activity of employees in the workplace (Lichtenthaler and Fischbach, 2018; Bakker and Oerlemans, 2019); similarly, we suggest that RC is also a kind of self-regulatory activity of job seekers in the process of finding reemployment and may mediate the relationship between JSC and RQ. Hulshof et al. (2020a) argued that RC, which is different from proactive job search behaviors (e.g., making a personal resume, attending interviews) and logically precedes or complements job search behaviors, can make the job search journey more motivating, challenging and less hindering. Most of the studies on the mediation mechanisms underlying the relationship between JSC and job search outcomes have concentrated on the perspectives of job search intensity, networking behavior, and job search strategy (Wanberg et al., 2002; Kuron, 2020; Le and Lin, 2021). In fact, job search demands and resources are present during the job-hunting process (Hulshof et al., 2020b); however, few studies have examined how job seekers proactively shape these demands and resources to find a satisfactory and well-matched job after setting clear job search goals. Therefore, RC was introduced and tested as a mediator in this study, aiming to expand the mediation mechanism of the goal-performance relationship.

Job hunting is normally embedded in a specific context that contains both favorable and unfavorable factors (Boswell et al., 2012). Social cognitive theory states that there is a dynamic interplay between person, behavior and the environment in the self-regulatory process (Bandura, 1991), so individuals’ self-regulatory activities in job searches are bound to be conditioned and influenced by the contextual environment, and a supportive and CEn may exert different effects. Therefore, it is necessary to explore individuals’ job search activities in combination in the reemployment context to more accurately reveal the self-regulation mechanism in their job searches.

**FIGURE 1:**
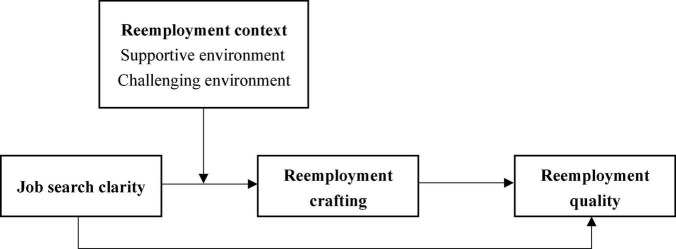
Proposed research model.

In summary, the aim of this study was to investigate the mediating effect of RC on the relationship between JSC and RQ among migrant workers and to examine the moderating effects of reemployment contexts involving supportive or CEns. The proposed research model is summarized in Figure 1. Our study makes the following contributions to the literature. First, in addition to the four mediating factors proposed by Goal-Setting Theory to explain why specific, high goals can increase an individual’s performance (Latham and Locke, 1991). Our study provides new insight into and an integrated explanation of the mediation mechanism of the goal-performance relationship from the perspective of self-regulation and expands Goal-Setting Theory by empirically testing the mediating role of RC on the relationship between JSC and RQ. Second, current study has examined the relationships among RC and environmental exploration, networking, reemployment chance (Hulshof et al., 2020a), while our study contributes to enriching the current literature on the antecedents and consequences of RC. Third, we investigated the reemployment context as the boundary condition and examined the independent and joint moderating effects of supportive and CEns on the internal mechanism of JSC on RQ via RC, which contributes to providing an in-depth understanding of when RC occurs in the reemployment context and expands the moderation mechanism of the goal-performance relationship.

## Theory and hypotheses

### Job search clarity and reemployment quality

RQ in this study refers to the extent to which migrant workers feel satisfied and matched with the new jobs they find after unemployment. We assume that JSC is positively associated with RQ among migrant workers. First, job seekers with high JSC normally have a clear idea of the type of job they desire and how to find a job and tend to adopt exploratory or focused job search strategies (Kuron, 2020), which are more effective and beneficial for job seekers not only to obtain more accurate employment information (Koen et al., 2010) but also to develop the right perception of person-job fit (Affum-Osei et al., 2021), thereby having positive impacts on job search outcomes (Crossley and Highhouse, 2005; Saks, 2006). In contrast, job seekers with low JSC tend to adopt a haphazard job search strategy, spend more time exploring options and search less intensely (Kuron, 2020), reducing the chance of finding reemployment. Moreover, due to the blurred concept of a desired job, Bao and Luo (2015), argued that job seekers with low JSC cannot judge which job is most suitable for them and cannot make the right employment decision from the various job opportunities; consequently, they are more likely to accept the first job offered to them or to take jobs that do not fit their interests (Wanberg et al., 2002). Second, JSC has the motivational function of helping job seekers with higher clarity be more motivated and engaged job search behaviors (Zikic and Saks, 2009; Guerrero and Rothstein, 2012; Bao and Luo, 2015) and thereby helps them obtain more job interviews and job offers (Saks and Ashforth, 2000; Côté et al., 2006), which enable the unemployed to choose a satisfying and well-matched job from numerous reemployment opportunities.

Therefore, we propose Hypothesis 1:

***H1:** JSC is positively related to* RQ.

### The mediating role of reemployment crafting

RC, which is the extension and application of job crafting in the field of reemployment, enables the unemployed to shape or create necessary environmental conditions to perform optimally in their job searches (Hulshof et al., 2020a). There are three types of RC: seeking resources (SR), which may include proactive behaviors such as seeking advice on how to apply for a specific job; seeking challenging demands (SCD), which may include proactive behaviors such as learning how to apply for a job online; and reducing hindering demands (RHD), which may include behaviors such as not thinking about job hunting for a week. As mentioned above, job search is a dynamic self-regulatory process (Kanfer et al., 2001); therefore, job crafting, as a kind of self-regulatory behavior in the job search process, may be an important path connecting job search goals and performance; that is, it may mediate the relationship between JSC and RQ.

Based on the self-regulation process as well as the motivating and orienting roles of job search goals, we suggest that JSC may be an antecedent factor that triggers and influences RC. First, according to self-regulation theory, the self-regulation process generally consists of three phases: forecasting, performance monitoring and self-reflection. The forecasting phase includes activities such as goal setting and strategy design; the performance monitoring phase includes activities of focusing on the task, making maximum efforts, trying specific strategies, and asking for help; and the third phase of self-reflection involves adaptive/defensive inferences that produce adaptive responses (e.g., changing the goal level, choosing other more effective strategies) or defensive responses (e.g., avoiding the task) (Bandura, 1986). Therefore, the process from clarifying job search goals to undertaking RC is a self-regulatory process in which clarifying job search goals corresponds to the activities in the forecasting phase of self-regulation, while RC corresponds to the activities that occur in the latter two stages of performance monitoring and self-reflection. Therefore, in accordance with the sequence of the self-regulation process, we postulate that JSC logically precedes and influences RC.

Second, based on the motivating role of goals, only setting clear, important and meaningful job search goals can have a motivational effect according to goal-setting theory (Locke and Latham, 2006). In addition, high JSC also means that job seekers tend to have a clear idea of the gap between themselves and their goals. While the primary purpose of job crafting is to acquire work meaning, the gap between person and environment is one of the drivers that motivates employees to enhance person-job fit through job crafting (Tims et al., 2016). Thus, we postulate that JSC promotes the motivations of the unemployed to implement RC to achieve their job search goals and narrow the gap between themselves and their goals. Moreover, in terms of the orienting role of goals (Kuron, 2020), based on goal orientation theory, proposed that JSC comprises the job search clarity-process (JSC-P), which is the clarity of the behaviors and strategies needed to successfully achieve job search goals and is learning-goal oriented, and job search clarity-outcome (JSC-O), which means the clarity of the requirements of the target job and is performance-goal oriented. Studies have indicated that individuals with a learning-goal orientation tend to not only increase the challenge level of their goals but also focus on the development of their competencies and skills, whereas individuals with a performance-goal orientation are more concerned with demonstrating personal competence (Dweck and Leggett, 1988; Van Yperen, 2003). Accordingly, Kuron (2020) further argued that JSC-P can elicit individuals’ exploratory job search strategies, including the behavioral actions of examining potential employment options (Crossley and Highhouse, 2005), SR such as consulting others about their job searches, and gathering information related to their career development (Koen et al., 2010). Hulshof et al. (2020a) also found that the relationship between environmental exploration (i.e., gathering information about jobs, careers, and organizations) and RC was reciprocal; seeking challenges not only influences environmental exploration but also affects RC behavior. Thus, JSC is assumed to elicit exploratory behaviors in job search, which in turn enhances RC. Regarding RHD, previous studies found that JSC was positively related to high self-efficacy and proactive personality (Zikic and Saks, 2009; Fort et al., 2011; Zhu et al., 2021), so migrant workers with high JSC are less likely to adopt defensive strategies such as RHD.

Therefore, we propose Hypothesis 2:

***H2:** JSC is positively related to (a) SR and (b) SCD and not related to (c) RHD.*

Hulshof et al. (2020a) stated that RC, which functions similarly to job crafting, is positively correlated with performance, and their study found that RC can enhance the intensity and quality of job search networks and eventually increase the chance of finding reemployment. Additionally, previous studies have also demonstrated that job crafting can bring about other positive outcomes for employees, such as job satisfaction and person-job fit (Tims et al., 2013; Tims et al., 2015). Similarly, we believe that RC is positively correlated with the reemployment outcomes of finding a satisfying and matching job. SR, such as asking others and unemployment agencies for advice, can enable the unemployed to obtain more information about jobs and careers, promote pre-entry person-job fit (i.e., person-job fit before being employed), predict post-entry person-job fit (i.e., person-job fit after being employed) (Saks and Ashforth, 2002), and help job seekers choose the right job offer. SCD, such as learning new things, making more effort in job search, and raising the job search bar, can help improve personal competencies and skills, reduce the gap between individuals and their goals, and maintain engagement in job search. These positive results from SR and SCD can ultimately increase the chances of finding a satisfactory and well-matched job. RHD, which can help alleviate health impairment (Petrou et al., 2012), however, has been reported to have little impact on finding reemployment (Hulshof et al., 2020a) or performance (Rudolph et al., 2017).

Therefore, we propose Hypothesis 3:

***H3:** Both (a) SR and (b) SCD are positively correlated with* RQ *in terms of finding a satisfactory and well-matched job, but (c) RHD is not.*

Given the above, unemployed migrant workers with high JSC will be motivated to implement RC of SR and SCD to pursue their job search goals, to close the gap between themselves and their goals, and to back up the exploration in their job searches, which can help maintain job search motivation and promote the pre-entry fit between individuals and their desired jobs, ultimately enhancing the chances of finding reemployment they are satisfied and matched with.

Therefore, we propose Hypothesis 4:

***H4:** (a) SR and (b) SCD mediate the relationship between JSC and* RQ, *but (c) RHD does not.*

### The moderating role of reemployment context

The reemployment context, which is the specific job search conditions or environment containing external job search resources and demands for individuals seeking reemployment, may have significant effects on the job search process and outcomes. The reemployment context is similar to the employment context, which contains supportive factors for individuals, such as financial support and employment counseling services, as well as challenging factors, such as high recruitment requirements and insufficient job vacancies (Song and Chathoth, 2008). There is interplay among personal, behavioral and environmental factors in the self-regulatory job search process, according to social cognitive theory (Bandura, 1991); thus, the relationship between JSC and RC, the process from goal setting to implementation, may be moderated by the reemployment context. As H2c proposed, the relationship between JSC and RHD may be not significant and will not be discussed below.

SEns, which can provide information, financial and psychological resources that the unemployed need in their job search, increase the subjective estimate of the probability of reemployment success according to achievement motivation theory (McClelland et al., 1976) and thereby will promote the achievement motivation of the unemployed with high JSC, making them more proactive and likely to select more challenging goals or tasks (Spence et al., 1989). Chen et al. (2020) pointed out that SR and SCD are closely associated with the fact that accomplishing challenging goals or tasks often requires more resources for support; therefore, individuals are more likely to seek and increase their resources to ensure the successful achievement of challenging goals or tasks. In addition, previous studies have demonstrated that the sense of autonomy and control motivates individuals to adopt a learning-goal orientated strategy (Wallhead and Ntoumanis, 2004) and can significantly strengthen the positive effect of learning-goal orientation (O’Keefe et al., 2013; Benita et al., 2014), while SEns increase individuals’ sense of autonomy and control according to self-determination theory (Ryan and Deci, 2017). Therefore, SEns not only encourage the unemployed to adopt a learning-goal oriented strategy in their job searches, such as by increasing the challenge of goals or tasks, focusing on learning new things and asking others for advice to develop their competencies and skills, but also make learning-goal orientation more effective. Moreover, Kim et al. (2018) found that SEns facilitate employees’ engagement in job crafting and similarly provide resources and favorable conditions for the unemployed to engage in RC.

Therefore, we propose Hypothesis 5:

SEn *moderates (a) the relationship between JSC and SR and (b) the relationship between JSC and SCD. That is, JSC elicits more SR and SCD for those in highly* SEn*s.*

The decline in employment availability brought about by impacts such as the pandemic has led to increasing competition for employment in the labor market. Employers also place higher demands on job seekers in terms of professional knowledge, skills, abilities, and experience, causing psychological tension in job seekers. Individuals with higher JSC typically have higher levels of job search self-efficacy (Fort et al., 2011; Travers et al., 2015; Ye et al., 2017), and they are more likely to perceive high demands and difficulties as challenges (Peng et al., 2015). Hulshof et al. (2020a) argued that SCD is an effective strategy for the unemployed to maintain their motivation (i.e., stay engaged) in their job searches, and the results from a meta-analysis study showed that challenging job demands are positively associated with work engagement (Crawford et al., 2010). Thus, unemployed migrant workers with high JSC tend to perceive difficulties or barriers in job search as challenges and may expend more effort and seek job search resources in response. Moreover, clear and specific goals generally strengthen goal commitment (Wright and Kacmar, 1994), which makes individuals more likely to try and persevere when encountering challenging situations. Feng (2016) noted that individuals with clear job search goals have increased goal commitment and do not easily give up when facing setbacks but instead exhibit more job search behaviors. Hence, CEns are expected to provoke SR and SCD for migrant workers with high JSC.

Therefore, we propose Hypothesis 6:

CEn *moderates (a) the relationship between JSC and SR and (b) the relationship between JSC and SCD. That is, JSC elicit more SR and SCD for those in highly* CEn*s.*

Studies related to job demands and resources have demonstrated that employees can more effectively transform high job resources into high performance only through challenging demands (Lewig et al., 2007; Bakker et al., 2010). Therefore, based on H4, H5, and H6, we further propose that the mediation effects of SR and SCD on the association between JSC and RQ may be greater highly supportive and CEns than for less supportive and CEns. That is, when the reemployment context is both supportive and challenging, individuals with high JSC are more likely to seek resources and challenging demands, which further leads to more chances to find a satisfactory and well-matched job.

Therefore, we propose Hypothesis *7:*

*The indirect effects of JSC on* RQ *via (a) SR and (b) SCD are significantly greater in highly supportive and* CEn*s.*

## Materials and methods

### Participants

In total, 295 migrant workers from the Yangtze River Delta of China were recruited to participate in this study. Of the participants, there were 173 males and 122 females, with an average age of 35.88 years old (SD = 8.40), and 56% of them had a senior high school education or above. The inclusion criteria were as follows: (a) being unemployed; (b) currently looking for a job; (c) access to online surveys using mobile phones and (d) voluntarily consenting to participate in this study. The participants received five Chinese yuan (approximately 0.75 US dollars) as a token of appreciation after each survey.

### Procedure

This study had a three-wave design and was conducted from February through July 2022, and permission to conduct this study was obtained from the university ethics committee before starting the investigation (Approval no. 2021057). We posted recruitment notices in the employment agencies and labor markets of seven cities after approval from marketing managers. These notices described the aims, requirements, risks, benefits and process of the study and provided a QR code to access the WeChat group for the survey. The data were then collected using an online survey link via the WeChat group, participation was voluntary, and the participants were assured that their responses would be confidential. In the first phase, a total of 533 migrant workers who were unemployed and looking for jobs consented to participate and completed a survey on JSC, reemployment context, reemployment status, and demographic variables. One month after the first survey, 502 of 533 initial respondents, for a 94.18% response rate, completed the survey of RC in the second phase. The third survey was conducted 3 months later, and only 295 of 502 migrant workers, for a 58.76% response rate, claimed to have found a new full-time job and completed the survey of reemployment satisfaction and person-job fit in the third phase. There were 23 observations with missing values, and missing data were replaced by the mean of the other existing observations of the same dimension.

### Measures

The scales were originally developed in English, translated into Chinese and then back translated into English. Two doctoral students majoring in English were invited to compare the back translations with the original versions. Based on their suggestions and the differences in culture and expression habits between Chinese and Westerners, some items were modified to ensure clarity, accuracy, and cultural adaptation. Moreover, all items were rated on a five-point scale ranging from 1 (does not apply to me) to 5 (totally applies to me).

#### Job search clarity

JSC was measured using a five-item scale developed by Côté et al. (2006), which has been shown to have good reliability and validity (the reported Cronbach’s α was 0.82) in China (Ye et al., 2017). An example item is and validity (the reported Cronbach Based on to have,” and Cronbach’s α for the scale in this study was 0.83.

#### Reemployment context

The contextual support and barrier scale developed by Song and Chathoth (2008) comprises two subscales of contextual support (six items) and contextual barriers (12 items) and was adopted to assess supportive and challenging job search environments, respectively. The reported Cronbach’s α for contextual support and contextual barrier were 0.88 and 0.93, respectively, in China (Liu and Zhang, 2017). Sample items for the SEn are rtive environmeto career counseling on how to pursue a jobvironmente and contextual barrier were 0.88 and 0.93on their sugge.” Cronbach’s α for the SEn in this study was 0.80. The subscale of contextual barriers was slightly modified to measure CEns. We combined the statements about computer skills, Mandarin skills, and foreign language skills in three different items into one item about professional skills. Sample items for CEns include “My professional skills are insufficient according to my potential employers” and “There are not enough vacant job positions for me.” Cronbach’s α for the CEn in this study was 0.82.

#### Reemployment crafting

We adopted the RC scale by Hulshof et al. (2020a) to assess the extent to which migrant workers implemented RC in the job search process over the past month. There were three types involving SR (three items), SCD (four items), and RHD (three items). Example items include “I have asked others for advice,” “I have undertaken more activities to find a job than I had planned,” and “I have tried to ensure that my quest for work is emotionally less intense.” Cronbach’s α for the three types was 0.72, 0.70, and 0.81, respectively, and Cronbach’s α for the overall scale was 0.73.

#### Reemployment quality

RQ was measured with person-job fit and reemployment satisfaction. We used the four-item scale from Saks and Ashforth (1997) to measure the level of fit between migrant workers and their current jobs. The Cronbach’s α for the scale has been reported to be 0.87 in Chinese participants (Xin et al., 2020). An example item is rticipants (migrant workers and their current jobs. The Cronbachand the differences in ‘s α for the scale in this study was 0.88. We used one item, e scale in am satisfied with my new job,” to measure reemployment satisfaction, and Cronbach’s α for the RQ survey in this study was 0.85.

#### Control variables

Gender, age, and education and length of unemployment have commonly been used in studies related to JSC, job search behavior and job search outcomes (Kanfer et al., 2001; Wanberg et al., 2002). Similar to previous studies, we controlled for gender, age, education, and months unemployed in this study.

### Analytical strategy

All data were self-reported, so Harman’s one-factor test (Harman, 1976) was adopted to assess the potential influence of common method bias using exploratory factor analysis (Podsakoff et al., 2003) and CFA. In addition, we tested the discriminant validity of our measures following previous studies (e.g., Iverson and Maguire, 2000; Xin et al., 2020), as well as the non-response bias (Lambert and Harrington, 1990) before testing the hypotheses. Moreover, to test the first-stage moderated mediation model, we followed (Preacher et al., 2010) and used model 4 from SPSS macro PROCESS (Hayes, 2013) to test the mediation effect of RC on the relationship between JSC and RQ first, and then tested the moderation effect of reemployment context using model 9. We also conducted simple-slope analysis to probe interaction effects as Aiken et al. (1991) suggested.

## Results

### Testing common method bias and non-response bias

The results of exploratory factor analysis extracted eight factors that accounted for 65.02% of the variance, and the first factor accounted for only 16.44%, less than the critical value of 40% (Podsakoff et al., 2003). Moreover, we tested for common method bias with a single-factor measurement model by combining all items into a single factor (Dedahanov et al., 2016). As shown in Table 1, the indices of the single-factor model indicate a poor model fit. These findings demonstrated that common method bias was unlikely to be a significant issue in this study. Moreover, non-response bias was tested using independent sample *t*-test (Lambert and Harrington, 1990), the results showed that the participants who dropped out had slightly lower scores on T1 SEn (*d* = 0.67, *t* = 1.98, *P* < 0.05), however, there were no differences on all other study variables and control variables (*P* > 0.05), indicating that dropout was completely random and non-response bias was not significant in our study.

**TABLE 1:** Model comparison.

Model	X^2^	*df*	X^2^/*df*	*RMSEA*	*CFI*	*TLI*	△X^2^	△*df*
Five-factor model	1193.35	584	2.04	0.06	0.90	0.89		
Four-factor model	1598.17	588	2.72	0.08	0.79	0.77	404.82***	4
Three-factor model 1	1982.77	591	3.35	0.09	0.68	0.66	789.42***	7
Three-factor model 2	1820.39	591	3.08	0.08	0.73	0.70	627.04***	7
Two-factor model	2203.63	593	3.72	0.10	0.62	0.59	1010.28***	9
Single-factor model	2864.81	594	4.82	0.12	0.32	0.28	1671.46***	10

***P < 0.001.

### Testing discriminant validity

The discriminant validity of each measure was also tested using confirmatory factor analysis (CFA), and we found that the five-factor model was superior to the other models, with a chi-square-to-degrees-of-freedom ratio (χ^2^/*df*) = 2.04, comparative fit index (CFI) = 0.90, Tucker–Lewis Index (TLI) = 0.89, and root mean square error of approximation (RMSEA) = 0.06 (Table 1), demonstrating good discriminative validity among the measures (Byrne, 2013; Wen et al., 2018).

Five-factor model: JSC, supportive environment (SEn), challenging environment (CEn), reemployment crafting (RC), reemployment quality (RQ).

Four-factor model: JSC, SEn + CEn, RC, RQ.

Three-factor model 1: JSC + SEn + CEn, RC, RQ.

Three-factor model 2: JSC, SEn + CEn, RC + RQ.

Two-factor model: JSC + SEn + CEn, RC + RQ.

Single-factor model: JSC + SEn + CEn + RC + RQ.

### Descriptive statistics and correlation analysis

Table 2 reports the means, standard deviations, and interrelations of all the variables. The results showed that JSC was significantly related to SR (*r* = 0.22, *P* < 0.001), SCD (*r* = 0.24, *P* < 0.01), and RQ (*r* = 0.24, *P* < 0.001). Both SR and SCD were significantly correlated with RQ (*r* = 0.27 and 0.28, respectively; *P* < 0.001). In addition, neither JSC nor RQ was significantly correlated with RHD (*r* = 0.05 and 0.08, respectively; *P* > 0.05). These findings preliminarily supported H1–H3.

**TABLE 2:** Means, standard deviation, and correlations for variables.

Variables		Means	*SD*	1	2	3	4	5	6	7	8	9	10
1	Gender	1.41	0.49	−									
2	Age	35.88	8.40	0.02	−								
3	Education	1.70	0.70	0.06	−0.15**	−							
4	Months unemployed	2.53	0.91	0.01	0.16**	–0.01	−						
5	JSC (T1)	16.71	3.18	–0.07	0.05	0.05	–0.06	−					
6	Supportive environment (T1)	19.39	3.49	0.04	0.08	0.07	0.06	0.26**	−				
7	Challenging environment (T1)	29.85	5.17	<0.01	> −0.01	–0.08	–0.04	−0.24**	–0.04	−			
8	SR (T2)	9.29	1.91	0.04	0.08	0.07	0.04	0.22**	0.19**	–0.07	−		
9	SCD (T2)	13.59	2.14	–0.08	0.04	0.09	0.04	0.24**	0.15**	−0.16**	0.51**	−	
10	RHD (T2)	10.68	1.56	0.11	0.13*	–0.06	0.03	0.05	0.01	0.06	0.07	–0.01	−
11	Reemployment quality (T3)	16.88	2.45	–0.04	0.03	0.03	−0.13*	0.18**	0.10	–0.06	0.24**	0.26**	0.01

*N* = 295. *P < 0.05. **P < 0.01.

Gender: male = 1, female = 2. Education: junior high school or below = 1, senior high school = 2, above senior high school = 3. Months unemployed: unemployed ≤ 1 month = 1, 1 month < unemployed ≤ 3 months = 2, 3 months < unemployed ≤ 6 months = 3, unemployed > 6 months = 4. JSC, job search clarity; SR, seeking resources; SCD, seeking challenging demands; RHD, reducing hindering demands.

### Testing mediation effects

Model 4 from SPSS macro PROCESS (Hayes, 2013) was used to test the mediation model. As shown in Table 3, the direct effect of JSC on RQ was positive and significant in the absence of the mediator (β = 0.22, *P* < 0.001), and H1 was supported. When RC was included, JSC was positively associated with SR (β = 0.22, *P* < 0.001) and SCD (β = 0.26, *P* < 0.001), supporting H2a and H2b, which in turn were positively associated with RQ (β = 0.19 and 0.18, respectively, *P* < 0.05), supporting H3a and H3b, while the relation between JSC and RQ became non-significant (β = 0.13, *P* > 0.05). As H2c and H3c proposed, the effect of JSC on RHD was not significant (β = 0.04, *P* > 0.05), nor was the effect of RHD on RQ (β = 0.003, *P* > 0.05), supporting H2c and H3c.

**TABLE 3:** The mediation model of reemployment crafting between JSC and reemployment quality.

Variable	Reemployment quality	Reemployment crafting			Reemployment quality
			
	*β* (*SE*)	SR	SCD	RHD	*β* (*SE*)
				
		*β* (*SE*)	*β* (*SE*)	*β* (*SE*)	
Constant	15.12***(1.15)	5.68***(0.89)	10.37***(0.99)	9.12***(0.74)	12.14***(1.54)
Gender	−0.12(0.29)	0.21 (0.22)	−0.31(0.25)	0.37 (0.18)	−0.10(0.28)
Age	0.01 (0.02)	0.02 (0.01)	0.01 (0.01)	0.02 (0.01)	0.01 (0.02)
Education	0.08 (0.20)	0.17 (0.16)	0.27 (0.18)	−0.12(0.13)	< 0.01(0.20)
Months unemployed	−0.33*(0.16)	0.09 (0.12)	0.11 (0.13)	0.02 (0.10)	−0.37*(0.15)
JSC	0.22***(0.07)	0.22***(0.06)	0.26***(0.06)	0.04 (0.05)	0.13 (0.07)
SR					0.18*(0.08)
SCD					0.19*(0.07)
RHD					< 0.01(0.09)
*R* ^2^	0.05	0.06	0.07	0.03	0.11
*F*	3.04*	3.88**	4.45***	2.05	4.59***

*N* = 295. *β*, standardized coefficient; SE, standardized error.

*P < 0.05. **P < 0.01. ***P < 0.01.

Next, to further confirm the mediation effects of RC, this study adopted the biased-corrected bootstrapping method developed by Preacher and Hayes (2008) to test mediation effects. The results of bootstrapping, as presented in Table 4, showed that the estimated direct effect of JSC on RQ was 0.13, and the 95% CI ranged from −0.01 to 0.28 (including zero). The mediation effects of SR and SCD were 0.04 and 0.05, respectively, and none of their 95% CIs crossed zero; thus, H4a and H4b were supported. However, the mediation effect of RHD was less than 0.01, and the 95% CI ranged from −0.01 to 0.02 (including zero), supporting H4c. RC fully mediated the relation between JSC and RQ, and the indirect effects accounted for 39.88% of the total effect.

**TABLE 4:** Mediation effects of reemployment crafting on the relationship between JSC and reemployment quality.

Path	Estimate	SE	95% CI
JSC → reemployment quality	0.13	0.07	[−0.01, 0.28]
JSC → SR → reemployment quality	0.04*	0.03	[0.002, 0.10]
JSC → SCD → reemployment quality	0.05*	0.03	[0.006, 0.11]
JSC → RHD → reemployment quality	<0.01	0.01	[−0.01, 0.01]
Total indirect effect	0.09**	0.03	[0.04, 0.17]
Total effect	0.22**	0.07	[0.07, 0.37]

*N* = 295. Bootstrap sample size = 5000.

CI, confidence interval.

*P < 0.05. **P < 0.01.

### Testing moderated mediation effects

According to Hayes (2013), model 9 from SPSS macro PROCESS was applied to test the moderated mediation model with RC as the mediator and reemployment context as the moderator. The results of the moderation test, as presented in Table 5, showed that when predicting SR, the interaction of JSC and SEn was significant and positive (β = 0.03, *P* < 0.05), as was the interaction of JSC and CEn (β = 0.04, *P* < 0.01, respectively). H5a and H6a were supported. In terms of SCD as the dependent variable, the interactions of JSC and SEn and JSC and CEn were also significant and positive, respectively (β = 0.05, *P* < 0.001; β = 0.04, *P* < 0.001, respectively), supporting H5b and H6b.

**TABLE 5:** Moderation effects of reemployment contexts on the relationship between JSC and reemployment crafting.

Variable	Reemployment crafting					
	
	SR		SCD		RHD	
			
	*B*	*SE*	β	*SE*	β	*SE*
Constant	7.89***	0.67	12.97***	0.74	9.45***	0.58
Gender	0.24	0.22	–0.28	0.25	039*	0.19
Age	0.02	0.01	0.11	0.24	0.02*	0.01
Education	0.11	0.15	0.21	0.25	−0.12	0.13
Months unemployed	0.09	0.12	0.10	0.13	0.03	0.10
JSC	0.22***	0.06	0.24***	0.07	0.06	0.05
Supportive environment	0.07*	0.03	0.05	0.04	−0.01	0.03
Challenging environment	–0.01	0.02	–0.05	0.03	0.02	0.02
JSC × supportive environment	0.03**	0.01	0.05***	0.01	<0.01	0.01
JSC × challenging environment	0.04***	0.01	0.04**	0.01	0.01	0.01
*R* ^2^	0.13		0.14		0.04	
*F*	4.88***		5.45***		0.47	

*N* = 295.

*P < 0.05. **P < 0.01. ***P < 0.01.

**FIGURE 2:**
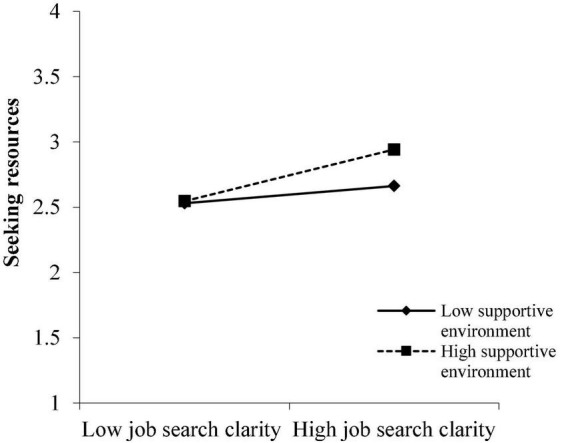
Moderation effect of supportive environment on the relationship between JSC and SR.

**FIGURE 3:**
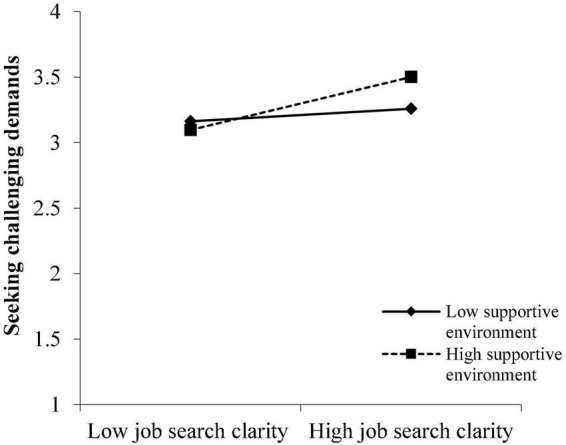
Moderation effect of supportive environment on the relationship between JSC and SCD.

To better comprehend the moderation of the reemployment context, simple slope analysis was conducted (Aiken et al., 1991). Figures 2, 3 illustrate that JSC had stronger and significant effects on SR (simple slope = 0.20, SE = 0.05, *P* < 0.001) and SCD (simple slope = 0.20, SE = 0.04, *P* < 0.001) for those with a high level of SEn (1 SD above the mean), while the effect of JSC on SR (simple slope = 0.07, SE = 0.05, *P* > 0.05) as well as on SCD (simple slope = 0.05, SE = 0.04, *P* > 0.05) was not significant for those with a low level of SEn (1 SD below the mean).

**FIGURE 4:**
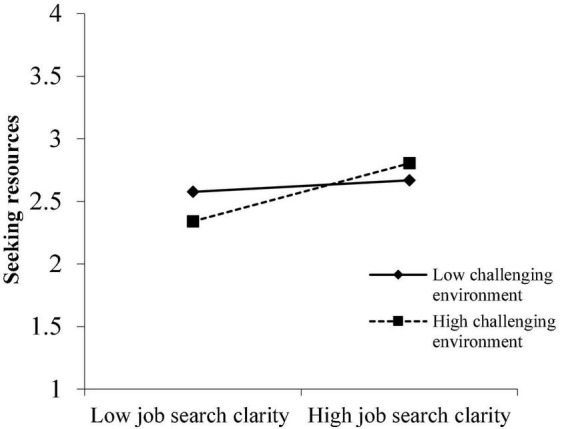
Moderation effect of challenging environment on the relationship between JSC and SR.

**FIGURE 5:**
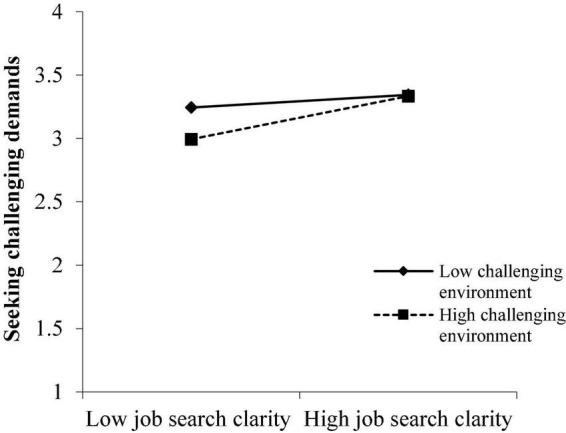
Moderation effect of challenging environment on the relationship between JSC and SCD.

Figures 4, 5 illustrate similar results: highly CEns exaggerated the effect of JSC on SR (simple slope = 0.22, SE = 0.03, *P* < 0.001) and on SCD (simple slope = 0.18, SE = 0.03, *P* < 0.001), while the relationships between JSC and SR (simple slope = 0.05, SE = 0.03, *P* > 0.05) and between JSC and SCD (simple slope = 0.05, SE = 0.03, *P* > 0.05) were weaker and not significant in low-level CEns.

Moreover, the results shown in Table 6 indicate that the indirect effect of JSC on RQ via RC differed as a function of reemployment context. That is, the mediation effect of SR (estimate = 0.10, SE = 0.05, 95% CI = 0.02, 0.23) as well as SCD (estimate = 0.12, SE = 0.05, 95% CI = 0.03, 0.24) was significant and greater when supportive and CEns were both at high levels, while it was not statistically significant when both were at low levels. The findings demonstrate that the reemployment context has a positive moderating effect on the mediating effect of RC according to Hayes (2015), providing support for H7. Additionally, we found that the mediation effect of SR was not significant (estimate = 0.03, SE = 0.03) in environments with high support but low challenge, as the 95% CI included zero, and SCD was not significant (estimate = 0.04, SE = 0.03) in environments with high challenge but low support, as the 95% CI included zero. In summary, the results provided strong support for the hypothesized moderating effect of the reemployment context.

**TABLE 6:** Conditional indirect effects of JSC on reemployment quality at different values of reemployment context.

Mediator	Supportive environment	Challenging environment	Estimate	SE	95% CI
SR	−1 SD	−1 SD	–0.02	0.03	[−0.10, 0.01]
	−1 SD	+1 SD	0.05*	0.03	[0.01, 0.12]
	+1 SD	−1 SD	0.03	0.03	[−0.004, 0.10]
	+1 SD	+1 SD	0.10*	0.05	[0.02, 0.23]
SCD	−1 SD	−1 SD	–0.02	0.02	[−0.09, 0.02]
	−1 SD	+1 SD	0.04	0.03	[−0.003, 0.12]
	+1 SD	−1 SD	0.04*	0.02	[0.01, 0.13]
	+1 SD	+1 SD	0.11*	0.05	[0.03, 0.24]

*N* = 295.

*P < 0.05.

Bootstrap sample size = 5000. +1 SD = 1 SD above the mean. −1 SD = 1 SD below the mean.

## Discussion

Using a three-wave design, the current study examined the effect of JSC on RQ with RC as a mediator and reemployment context as a moderator. Data collected from 295 migrant workers over three phases revealed that JSC had a predictive effect on RQ; SR and SCD fully mediated the relationship between JSC and RQ. Both supportive and CEns had moderating effects on the relationship between JSC and SR and the relationship between JSC and SCD. The mediation effects of SR and SCD on the relationship between JSC and RQ were significant and stronger when the supportive and challenging employment environments were both at high levels.

### Theoretical implications

There are several theoretical implications in our study. First, JSC has a positive direct effect on RQ, which suggests that migrant workers with high JSC are more likely to find a satisfactory and well-matched job. This is similar to findings reported in a previous study that JSC is positively correlated with employment status and employment quality (Wanberg et al., 2002; Zikic and Saks, 2009). However, we cannot conclude from our data that JSC can help migrant workers succeed in finding reemployment, as no differences in the T1 JSC scores were found between the participants who claimed to have found a job and dropouts who were still unemployed at the time of the third survey. What is certain, however, is that among those migrant workers who succeed in finding reemployment, the higher their JSC, the more satisfying and well-matched are the jobs they get. Our study provides empirical evidence for the relationship between JSC and migrant workers obtaining satisfactory and well-matched reemployment and enriches the literature on the effect of JSC.

Furthermore, previous studies have identified goal orientation, effort, persistence, and task strategy as mediation factors to explain the mechanisms of the goal-performance relationship (Latham and Locke, 1991). This study introduces RC into the relationship between JSC and RQ for the first time and finds that SR and SCD fully mediates the relationship between JSC and RQ. Our findings confirm the goal-oriented and motivating functions of JSC (Côté et al., 2006; Zikic and Saks, 2009; Kuron, 2020) and indicate that RC is also a kind of self-regulatory activity with a similar effect as job crafting in promoting person-job fit (Tims et al., 2016), support exploration in job search (Hulshof et al., 2020a), and ultimately leading to success in finding satisfactory and matching jobs. Our study provides new insight into and an integrated explanation of the mediation mechanism of the goal-performance relationship while extending the literature on the antecedents and consequences of RC. In addition, the result showed that RHD had no mediation effect on the relationship between JSC and RQ, which is in line with previous research findings, indicating that individuals with high JSC are more proactive and confident (Ye et al., 2017; Zhu et al., 2021) and less likely to adopt defensive strategies such as RHD, and it is also consistent with the findings that RHD helps individuals decrease the psychological impairment caused by stress but that it has little effect on positive outcomes (Petrou et al., 2012; Rudolph et al., 2017).

With regard to the moderating effects of reemployment context, the results in this study show that a SEn exaggerates the relationship between JSC and SR, as well as the relationship of JSC and SCD, highlighting the positive effect of SEns on job search. This is also similar to Hulshof et al.’s (2020a) finding that social support plays an important contextual role in RC. Moreover, CEns were found to have the same moderating effect as SEns. Although a previous study argued that hindrance may weaken crafting behaviors (Liu et al., 2019), whether the contextual barriers of job search for the individual are a challenge or hindrance partly depends on the individual’s personality. Individuals with high JSC typically have stronger self-efficacy (Fort et al., 2011); they are more likely to perceive contextual disadvantages as challenges (Peng et al., 2015) and challenging demands as able to provoke the motivation of crafting (Berg et al., 2010; Tims and Bakker, 2010). In summary, the findings in our study provide empirical support for the conclusions of existing studies and reconfirm the validity of social cognitive theory, that is, the existence of interactions among individuals’ cognitions, behaviors and the environment in job hunting.

Furthermore, we found that the mediating effect of SR and SCD were significant and stronger when both the supportive and CEns were at high levels. However, the mediating effect of SCD was not significant in high challenging but low SEns, and the mediating effect of SR was not significant in high supportive but low CEns, implying that supportive and CEns in the reemployment context can function not only independently but also in combination, and RC is a kind of self-regulation activity, similar to job crafting, that can offset what is lacking in the environment in terms of resources and demands, aiming to strike a balance between job search demands and resources. The results are similar to the findings of previous studies that high challenging job demands with the absence of job resources decrease the engagement of employees and bring about other negative outcomes, and high job resources exert more positive effects only in CEns (Bakker et al., 2007, 2010; Lewig et al., 2007). Moreover, compared with previous studies that concentrated on variables such as ability, goal commitment, feedback, and resource as the main moderators in the goal-performance relationship (Locke and Latham, 2006), our study not only demonstrated the positive role of resource support but also found the effect of challenging demands in the context and the combined effects of both, which expands the understanding of the moderation mechanism of the goal-performance relationship and enriches the existing research findings.

### Practical implications

To promote RQ among migrant workers, the following measures can be adopted.

First, our empirical results showed that JSC affects RC and RQ. Therefore, employment service agencies should provide migrant workers coaching and training for career planning so that they can establish correct views about careers and jobs; clarify their career goals, job search goals and the gap between their abilities, knowledge, skills and their goals; and make specific and feasible job search plans to achieve their goals.

Second, RC involving SR and SCD affects RQ. Hence, it is necessary to encourage migrant workers to carry out promotion-focused RC, such as by expanding and making good use of available job search resources, increasing their job search engagement, learning job search strategies and skills (e.g., employment information search, resumption design, interview skills), and developing their employability.

Third, RC increases the mediation effects of SR and SCD on the association between JSC and RQ. Therefore, the job search process should be specifically managed by alleviating the job-hunting stress of migrant workers, teaching them to rationally deal with the unfavorable factors in job search and regard the difficulties in job hunting as challenges, and minimizing the use of negative coping strategies. In addition, giving sufficient understanding and tolerance of the failure of migrant workers in job hunting and encouraging them to reflect on and learn from failures to grow from them. Furthermore, a reemployment support system should be built for migrant workers with the participation of multiple parties, including the government, families and non-profit organizations, to clarify the responsibilities and tasks of each party, taking advantage of the resources of multiple parties to provide comprehensive support including policy, financial, information, and psychological assistance.

Last but not least, in the post-COVID-19 era, the government should balance the relationship between security and development, adopt more scientific and accurate pandemic prevention measures to avoid excessive pandemic prevention in China, and focus on how to ensure the stable development of the national economy while preventing the spread of the pandemic. More jobs can be created to fundamentally solve the unemployment problem of migrant workers only through economic development.

### Limitations and future research

First, the measure of JSC did not distinguish between JSC-P (learning-goal oriented) and JSC-O (performance-goal oriented) in detail; the JSC scale we adopted contains both process and outcome clarity. Therefore, future studies should distinguish the influence mechanisms of these forms of goal-oriented JSC on migrant workers’ RQ via RC. Second, the sample of this study was relatively small and could not fully represent all migrant workers in China, and the moderated mediation model in this study was tested with the data from the participants who succeeded in finding reemployment; thus, the conclusion needs to be further tested in a broader population in the future. Third, based on the self-regulation process, we treated JSC as an antecedent variable of RC and examined how JSC affects RC, similar to the circular relations of the three phases in the self-regulation process. The relationship between JSC and RC can also be circular and reciprocal, and individuals may readjust their job search goals and set clearer goals after RC; therefore, this reciprocal relationship should be tested in future research. Fourth, given the impacts of the pandemic, including prevention policies, on job hunting, a potential bias in our results may exist and should be considered; for example, migrant workers may lower their job search expectations and thus be more likely to be satisfied with reemployment, and their job search activities involving crafting behaviors may be constrained by the pandemic and prevention policies. Therefore, the impacts of the pandemic should be included in the model of the job search process and outcomes in future studies.

## Conclusion

The present research demonstrated that RC is a proactive technique for the unemployed to optimize their job search performance by SR and challenging demands, which mediate the relationship between JSC and RQ. Furthermore, highly supportive and CEns strengthen the mediating effect of RC, thereby contributing to the understanding of how RC and the contextual environment function in the job search process. Therefore, in the post-COVID-19 era, which is full of difficulties and challenges in finding reemployment, interventions concentrated on setting clear job search goals and plans, providing reemployment support, and promoting RC are important to increase RQ.

## Data availability statement

The raw data supporting the conclusions of this article will be made available by the authors, without undue reservation.

## Ethics statement

The studies involving human participants were reviewed and approved by the Ethics Committee of Nanjing Tech University. The patients/participants provided their written informed consent to participate in this study.

## Author contributions

XL designed the research and wrote the manuscript. KZ and ZW carried the investigation and analyzed the research data. All authors contributed to the article and approved the submitted version.

## References

[B1] Affum-Osei E. Mensah H. K. Asante E. A. Forkuoh S. K. (2021). Evaluating a job search strategy model of fit perceptions: A construct validation amongst unemployed job seekers. *Career Dev. Int.* 26 269–289. 10.1108/CDI-09-2020-0249

[B2] Aiken L. S. West S. G. Reno R. R. (1991). *Multiple regression: Testing and interpreting interactions.* Newbury Park, CA: Sage Publication.

[B3] Bakker A. B. Oerlemans W. G. (2019). Daily job crafting and momentary work engagement: A self-determination and self-regulation perspective. *J. Vocat. Behav.* 112 417–430. 10.1016/j.jvb.2018.12.005

[B4] Bakker A. B. Hakanen J. J. Demerouti E. Xanthopoulou D. (2007). Job resources boost work engagement, particularly when job demands are high. *J. Educ. Psychol.* 99 274–284. 10.1037/0022-0663.99.2.274

[B5] Bakker A. B. Van Veldhoven M. Xanthopoulou D. (2010). Beyond the demand-control model: Thriving on high job demands and resources. *J. Pers. Psychol.* 9 3–16. 10.1027/1866-5888/a000006

[B6] Bandura A. (1986). *Social foundations of thought and action: A social cognitive theory.* Englewood Cliffs, NJ: Prentice-Hall, Inc.

[B7] Bandura A. (1991). Social cognitive theory of self-regulation. *Organ. Behav. Hum. Decis. Process.* 50 248–287. 10.1016/0749-5978(91)90022-L

[B8] Bao Z. Luo P. (2015). How college students’ job search self-efficacy and clarity affect job search activities. *Soc. Behav. Pers.* 43 39–51. 10.2224/sbp.2015.43.1.39

[B9] Benita M. Roth G. Deci E. L. (2014). When are mastery goals more adaptive? It depends on experiences of autonomy support and autonomy. *J. Educ. Psychol.* 106 258–267. 10.1037/a0034007

[B10] Berg J. M. Wrzesniewski A. Dutton J. E. (2010). Perceiving and responding to challenges in job crafting at different ranks: When proactivity requires adaptivity. *J. Organ. Behav.* 31 158–186. 10.1002/job.645

[B11] Boswell W. R. Zimmerman R. D. Swider B. W. (2012). Employee job search: Toward an understanding of search context and search objectives. *J. Manag.* 38 129–163. 10.1177/0149206311421829

[B12] Byrne B. M. (2013). *Structural equation modeling with Mplus: Basic concepts, applications, and programming.* New York, NY: Routledge. 10.4324/9780203807644

[B13] Chen J. A. Chen S. Chen R. (2020). An empirical study on achievement motivation and consistent culture as dual core drivers of self-oriented job-crafting behavior: Based on self-regulatory theory. *Manag. Rev.* 32 170–183.

[B14] Côté S. Saks A. M. Zikic J. (2006). Trait affect and job search outcomes. *J. Vocat. Behav.* 68 233–252. 10.1016/j.jvb.2005.08.001

[B15] Crawford E. R. Lepine J. A. Rich B. L. (2010). Linking job demands and resources to employee engagement and burnout: A theoretical extension and meta-analytic test. *J. Appl. Psychol.* 95 834–848. 10.1037/a0019364 20836586

[B16] Crossley C. D. Highhouse S. (2005). Relation of job search and choice process with subsequent satisfaction. *J. Econ. Psychol.* 26 255–268. 10.1016/j.joep.2004.04.001

[B17] Dedahanov A. T. Lee D. H. Rhee J. Yoon J. (2016). Entrepreneur’s paternalistic leadership style and creativity: The mediating role of employee voice. *Manag. Decis.* 54 2310–2324. 10.1108/MD-11-2015-0537

[B18] Dweck C. S. Leggett E. L. (1988). A social-cognitive approach to motivation and personality. *Psychol. Rev.* 95 256–273. 10.1037/0033-295X.95.2.256

[B19] Feng C. L. (2016). A longitudinal study on influences of job-search clarity on career development: Based on social cognitive career theory. *J. Shandong Norm. Univ.* 61 121–127.

[B20] Fort I. Jacquet F. Leroy N. (2011). Self-efficacy, goals, and job search behaviors. *Career Dev. Int.* 16 469–481. 10.1108/13620431111168886

[B21] Gao K. Qu Y. Wang S. Bai P. (2022). The impact of the COVID-19 pandemic on people’s health and management strategies. *Science* 10 158–164.

[B22] Guerrero L. Rothstein M. G. (2012). Antecedents of underemployment: Job search of skilled immigrants in Canada. *Appl. Psychol.* 61 323–346. 10.1111/j.1464-0597.2011.00470.x

[B23] Harman H. H. (1976). *Modern factor analysis.* Chicago, IL: University of Chicago Press.

[B24] Hayes A. F. (2013). *Introduction to mediation, moderation, and conditional process analysis.* New York, NY: The Guilford Press.

[B25] Hayes A. F. (2015). An index and test of linear moderated mediation. *Multivariate Behav. Res.* 50 1–22. 10.1080/00273171.2014.962683 26609740

[B26] Hulshof I. L. Demerouti E. Le Blanc P. M. (2020a). Reemployment crafting: Proactively shaping one’s job search. *J. Appl. Psychol.* 105 58–79. 10.1037/apl0000419 31192650

[B27] Hulshof I. L. Demerouti E. Le Blanc P. M. (2020b). A job search demands-resources intervention among the unemployed: Effects on well-being, job search behavior and reemployment chances. *J. Occup. Health Psychol.* 25 17–31. 10.1037/ocp0000167 31478707

[B28] Iverson R. D. Maguire C. (2000). The relationship between job and life satisfaction: Evidence from a remote mining community. *Hum. Relat.* 53 807–839. 10.1177/0018726700536003

[B29] Kanfer R. Wanberg C. R. Kantrowitz T. M. (2001). Job search and employment: A personality–motivational analysis and meta-analytic review. *J. Appl. Psychol.* 86 837–855. 10.1037//0021-9010.86.5.837 11596801

[B30] Kim H. Im J. Qu H. NamKoong J. (2018). Antecedent and consequences of job crafting: An organizational level approach. *Int. J. Contemp. Hosp. Manag.* 30 1863–1881. 10.1108/IJCHM-01-2017-0040

[B31] Koen J. Klehe U. C. Van Vianen A. E. Zikic J. Nauta A. (2010). Job-search strategies and reemployment quality: The impact of career adaptability. *J. Vocat. Behav.* 77 126–139. 10.1016/j.jvb.2010.02.004

[B32] Kuron L. (2020). *Clarifying job search clarity: Investigating job search as a self-regulatory process*. Ph.D. thesis. Waterloo, ON: Wilfrid Laurier University.

[B33] Lambert D. M. Harrington T. C. (1990). Measuring nonresponse bias in customer service mail surveys. *J. Bus. Logist.* 11 5–25. 12816142

[B34] Latham G. P. Locke E. A. (1991). Self-regulation through goal setting. *Organ. Behav. Hum. Decis. Process.* 50 212–247. 10.1016/0749-5978(91)90021-K

[B35] Le S. T. Lin S. P. (2021). Proactive personality and the job search outcomes: The mediating role of networking behaviour. *Brit. J. Guid. Couns.* 1–17. 10.1080/03069885.2021.1998362 [Epub ahead of print].

[B36] Lewig K. A. Xanthopoulou D. Bakker A. B. Dollard M. F. Metzer J. C. (2007). Burnout and connectedness among Australian volunteers: A test of the job demands–resources model. *J. Vocat. Behav.* 71 429–445. 10.1016/j.jvb.2007.07.003

[B37] Lichtenthaler P. W. Fischbach A. (2018). A meta-analysis on promotion-and prevention-focused job crafting. *Eur. J. Work Organ. Psychol.* 28 30–50. 10.1080/1359432X.2018.1527767

[B38] Liu C. Zhang B. (2017). An analysis about relationships among future work selves, job search clarity and job search behavior: The moderating role of contextual support and contextual barrier. *Hum. Resour. Dev. China* 12 60–72.

[B39] Liu X. G. Liu Y. C. Yan Y. (2022). China’s macro-economy in 2022 with growth stability as the first policy priority. *Econ. Theory Bus. Manag.* 42 4–22.

[B40] Liu Y. Yang D. T. An Y. R. (2019). Research on the relationship between challenging-hindering stress and work well-being: The mediating effect of job crafting. *Contemp. Econ. Manag.* 41 77–84.

[B41] Locke E. A. Latham G. P. (2006). New directions in goal-setting theory. *Curr. Dir. Psychol.* 15 265–268. 10.1111/j.1467-8721.2006.00449.x

[B42] McClelland D. C. Atkinson J. W. Clark R. A. Lowell E. L. (1976). *The achievement motive.* Irvington, NJ: Appleton-Century-Crofts.

[B43] Ming J. Wang M. L. (2015). Can job change effectively enhance employment quality of migrant workers? *China Soft Sci.* 12 49–62.

[B44] National Bureau of Statistics (2022). *2021 migrant worker monitoring survey report. Open government information.* Available online at: http://www.stats.gov.cn/xxgk/sjfb/zxfb2020/202204/t20220429_1830139.html (accessed April 29, 2022).

[B45] O’Keefe P. A. Ben-Eliyahu A. Linnenbrink-Garcia L. (2013). Shaping achievement goal orientations in a mastery-structured environment and concomitant changes in related contingencies of self-worth. *Motiv. Emot.* 37 50–64. 10.1007/s11031-012-9293-6

[B46] Peng A. C. Schaubroeck J. M. Xie J. L. (2015). When confidence comes and goes: How variation in self-efficacy moderates stressor–strain relationships. *J. Occup. Health Psychol.* 20 359–376. 10.1037/a0038588 25602277

[B47] Petrou P. Demerouti E. Peeters M. C. Schaufeli W. B. Hetland J. (2012). Crafting a job on a daily basis: Contextual correlates and the link to work engagement. *J. Organ. Behav.* 33 1120–1141. 10.1002/job.1783

[B48] Podsakoff P. M. MacKenzie S. B. Lee J. Y. Podsakoff N. P. (2003). Common method biases in behavioral research: A critical review of the literature and recommended remedies. *J. Appl. Psychol.* 88 879–903. 10.1037/0021-9010.88.5.879 14516251

[B49] Preacher K. J. Hayes A. F. (2008). *Assessing mediation in communication research.* London: The Sage sourcebook of advanced data analysis methods for communication research, 13–54. 10.4135/9781452272054.n2

[B50] Preacher K. J. Zyphur M. J. Zhang Z. (2010). A general multilevel SEM framework for assessing multilevel mediation. *Psychol. Methods* 15 209–233. 10.1037/a0020141 20822249

[B51] Rudolph C. W. Katz I. M. Lavigne K. N. Zacher H. (2017). Job crafting: A meta-analysis of relationships with individual differences, job characteristics, and work outcomes. *J. Vocat. Behav.* 102 112–138. 10.1016/j.jvb.2017.05.008

[B52] Ryan R. M. Deci E. L. (2017). *Self-determination theory: Basic psychological needs in motivation, development, and wellness.* New York, NY: Guilford Publications. 10.1521/978.14625/28806

[B53] Saks A. M. (2006). Multiple predictors and criteria of job search success. *J. Vocat. Behav.* 68 400–415. 10.1016/j.jvb.2005.10.001

[B54] Saks A. M. Ashforth B. E. (1997). A longitudinal investigation of the relationships between job information sources, applicant perceptions of fit, and outwork comes. *Pers. Psychol.* 50 395–426. 10.1111/j.1744-6570.1997.tb00913.x

[B55] Saks A. M. Ashforth B. E. (2000). Change in job search behaviors and employment outcomes. *J. Vocat. Behav.* 56 277–287. 10.1006/jvbe.1999.1714

[B56] Saks A. M. Ashforth B. E. (2002). Is job search related to employment quality? It all depends on the fit. *J. Appl. Psychol*. 87, 646–654. 10.1037/0021-9010.87.4.646 12184569

[B57] Song Z. Chathoth P. K. (2008). Career choice goals: The contribution of vocational interests, contextual support, and contextual barrier. *J. China Tour. Res.* 4 98–123. 10.1080/19388160802099923

[B58] Spence J. T. Pred R. S. Helmreich R. L. (1989). Achievement strivings, scholastic aptitude and academic performance: A follow-up to impatience versus achievement strivings in the type a pattern. *J. Appl. Psychol.* 74 176–178. 10.1037/0021-9010.74.1.176 2925558

[B59] Sun B. Y. (2022). *Stable development of market players, tamping the cornerstone of economic stability.* Available online at: http://news.china.com.cn/2022-02/06/content_78030775.htm (accessed October 3, 2022).

[B60] Tims M. Bakker A. B. (2010). Job crafting: Towards a new model of individual job redesign. *S. Afr. J. Ind. Psychol.* 36 1–9. 10.4102/sajip.v36i2.841

[B61] Tims M. Bakker A. B. Derks D. (2013). The impact of job crafting on job demands, job resources, and well-being. *J. Occup. Health Psychol.* 18 230–240. 10.1037/a0032141 23506549

[B62] Tims M. Bakker A. B. Derks D. (2015). Job crafting and job performance: A longitudinal study. *Eur. J. Work Organ. Psychol.* 24 914–928. 10.1080/1359432X.2014.969245

[B63] Tims M. Derks D. Bakker A. B. (2016). Job crafting and its relationships with person–job fit and meaningfulness: A three-wave study. *J. Vocat. Behav.* 92 44–53. 10.1016/j.jvb.2015.11.007

[B64] Travers C. J. Morisano D. Locke E. A. (2015). Self-reflection, growth goals, and academic outcomes: A qualitative study. *Br. J. Educ. Psychol.* 85 224–241. 10.1111/bjep.12059 25546509

[B65] Van Yperen N. W. (2003). Task interest and actual performance: The moderating effects of assigned and adopted purpose goals. *J. Pers. Soc. Psychol* 85:1006. 10.1037/0022-3514.85.6.1006 14674810

[B66] Wallhead T. L. Ntoumanis N. (2004). Effects of a sport education intervention on students’ motivational responses in physical education. *J. Teach. Phys. Educ.* 23 4–18. 10.1123/jtpe.23.1.4

[B67] Wanberg C. R. Hough L. M. Song Z. (2002). Predictive validity of a multidisciplinary model of reemployment success. *J. Appl. Psychol.* 87 1100–1120. 10.1037/0021-9010.87.6.1100 12558217

[B68] Wen Z. Huang B. Tang D. (2018). Preliminary work for modeling questionnaire data. *J. Psychol. Sci.* 41 204–210.

[B69] Wright P. M. Kacmar K. M. (1994). Goal specificity as a determinant of goal commitment and goal change. *Organ. Behav. Hum. Decis. Process.* 59 242–260. 10.1006/obhd.1994.1059

[B70] Xin L. Zhou W. Li M. Tang F. (2020). Career success criteria clarity as a predictor of employment outcomes. *Front. Psychol.* 11:540. 10.3389/fpsyg.2020.00540 32372998 PMC7176933

[B71] Ye B. J. Zheng Q. Dong S. H. Fang X. T. Liu L. L. (2017). The effect of calling on employability of college students: The mediating role of job searching clarity and job searching self-efficacy. *Psychol. Dev. Educ.* 33 37–44.

[B72] Zhu H. Zhang H. Tu A. Zhang S. (2021). The mediating roles of core self-evaluation and career exploration in the association between proactive personality and job search clarity. *Front. Psychol.* 12:609050. 10.3389/fpsyg.2021.609050 34149503 PMC8211878

[B73] Zikic J. Saks A. M. (2009). Job search and social cognitive theory: The role of career-relevant activities. *J. Vocat. Behav.* 74 117–127. 10.1016/j.jvb.2008.11.001

